# 3,3′-Diindolylmethane and indole-3-carbinol: potential therapeutic molecules for cancer chemoprevention and treatment via regulating cellular signaling pathways

**DOI:** 10.1186/s12935-023-03031-4

**Published:** 2023-08-26

**Authors:** Octavio Daniel Reyes-Hernández, Gabriela Figueroa-González, Laura Itzel Quintas-Granados, Stephany Celeste Gutiérrez-Ruíz, Hector Hernández-Parra, Alejandra Romero-Montero, María Luisa Del Prado-Audelo, Sergio Alberto Bernal-Chavez, Hernán Cortés, Sheila I. Peña-Corona, Lashyn Kiyekbayeva, Dilek Arslan Ateşşahin, Tamar Goloshvili, Gerardo Leyva-Gómez, Javad Sharifi-Rad

**Affiliations:** 1 Laboratorio de Biología Molecular del Cáncer, Facultad de Estudios Superiores Zaragoza, UMIEZ, Universidad Nacional Autónoma de México, Ciudad de México, 09230 Mexico; 2 Laboratorio de Farmacogenética, Facultad de Estudios Superiores Zaragoza, UMIEZ, Universidad Nacional Autónoma de México, Ciudad de México, 09230 Mexico; 3 Universidad Mexiquense del Bicentenario, Unidad de Estudios Superiores Tultitlán, Mexico, Mexico; 4 Departamento de Farmacia, Facultad de Química, Universidad Nacional Autónoma de México, Ciudad de México, 04510 Mexico; 5 Escuela de Ingeniería y Ciencias, Tecnologico de Monterrey, Campus Ciudad de México, C. Puente 222, Ciudad de México, 14380 Mexico; 6 Laboratorio de Medicina Genómica, Departamento de Genómica, Instituto Nacional de Rehabilitación Luis Guillermo Ibarra Ibarra, Ciudad de Mexico, Mexico; 7 Pharmaceutical School, Department of Pharmaceutical Technology, Asfendiyarov Kazakh National Medical University, Almaty, Kazakhstan; 8 Faculties of Pharmacy, Public Health and Nursing, Kazakh-Russian Medical University, Almaty, Kazakhstan; 9 Baskil Vocational School, Department of Plant and Animal Production, Fırat University, Elazıg, 23100 Turkey; 10 Department of Plant Physiology and Genetic Resources, Institute of Botany, Ilia State University, Tbilisi, 0162 Georgia; 11 Facultad de Medicina, Universidad del Azuay, Cuenca, Ecuador

**Keywords:** Chemotherapeutic drugs, Indole-3-carbinol (I3C), 3,3'-diindolylmethane (DIM), Cancer, Anti-tumor action

## Abstract

Dietary compounds in cancer prevention have gained significant consideration as a viable method. Indole-3-carbinol (I3C) and 3,3′-diindolylmethane (DIM) are heterocyclic and bioactive chemicals found in cruciferous vegetables like broccoli, cauliflower, cabbage, and brussels sprouts. They are synthesized after glycolysis from the glucosinolate structure. Clinical and preclinical trials have evaluated the pharmacokinetic/pharmacodynamic, effectiveness, antioxidant, cancer-preventing (cervical dysplasia, prostate cancer, breast cancer), and anti-tumor activities of I3C and DIM involved with polyphenolic derivatives created in the digestion showing promising results. However, the exact mechanism by which they exert anti-cancer and apoptosis-inducing properties has yet to be entirely understood. Via this study, we update the existing knowledge of the state of anti-cancer investigation concerning I3C and DIM chemicals. We have also summarized; (i) the recent advancements in the use of I3C/DIM as therapeutic molecules since they represent potentially appealing anti-cancer agents, (ii) the available literature on the I3C and DIM characterization, and the challenges related to pharmacologic properties such as low solubility, and poor bioavailability, (iii) the synthesis and semi-synthetic derivatives, (iv) the mechanism of anti-tumor action in vitro/in vivo, (v) the action in cellular signaling pathways related to the regulation of apoptosis and anoikis as well as the cell cycle progression and cell proliferation such as peroxisome proliferator-activated receptor and PPARγ agonists; SR13668, Akt inhibitor, cyclins regulation, ER-dependent-independent pathways, and their current medical applications, to recognize research opportunities to potentially use these compounds instead chemotherapeutic synthetic drugs.

## Introduction

Cancer is a significant public health issue and is the highest reason for death worldwide in countries of all income levels (low-, middle- and high-income countries) [1, 2]. In 2019 more than 10 million people perished by cancer, about twice the amount reported in 1990, according to data from the Global Burden Disease [3]. As a result, cancer deaths are expected to increase as populations age and develop styles of life that increase cancer risk, such as smoking, inactive lifestyles, and obesity [2].

Cancer pathogenesis is highly complex and is related to many mechanisms [4]. The progress against cancer has accelerated in recent years because of advancements in earlier detection, surgical procedures, and targeted treatments [1]. Several chemotherapeutic medications are available for cancer therapy. However, some have significant side effects, efficacies limited to specific patients, and are harmful and costly compared to traditional medicines. Therefore, developing, studying, and characterizing new anti-cancer medications with lower toxicity, price, and efficiency is challenging for researchers [4–6].

Patients’ diets are associated with prevention, evolution, advancement, and cancer treatment. Therefore, natural substances may be one possible aid for a new era of therapeutics for preventing and treating cancer [5]. For example, a more increased dietary intake of fruits and cruciferous vegetables is related to lower risk and prevents cancer evolution. In addition, natural chemicals in fruits, vegetables, and spices stop mechanisms implicated in the development of cancers and evoke tools related to the disease’s prevention [6, 7].

Research has indicated that indoles are heterocyclic compounds naturally found in many plants and are the bioactive component of cruciferous vegetables like broccoli, brussels sprouts, cauliflower, and others [6]. Since heterocyclic chemicals are utilized as hydrogen bond donors and acceptors, they could effectively attach to biological targets via intermolecular hydrogen bonds [5, 8, 9].

Today, we know many indole compounds with different activities and beneficial properties. For example, dietary indoles such as 3,3′-diindolylmethane (DIM)/Indole-3-carbinol (I3C) are robust prospects for chemotherapeutic chemicals. However, the exact action mechanism by which act to exert the anti-cancer and apoptosis-inducing properties has yet to be entirely understood. Therefore, the testable question in this study is: Could IC3 or DIM as a natural chemical origin be used instead of chemotherapeutic synthetic drugs? To know this, in this article, we analyze the current state of their characterization, derivatives, mechanism of anti-tumor action, and medical applications in animal models and clinical trials.

### Review methodology

A web-based review was completed from August to December 2022. Scopus, Google Scholar, and PubMed were used as research databases. We used the Medical Subject Headings (MeSH) for searching, or their combinations “3,3′-diindolylmethane (DIM), Indole-3-carbinol (I3C), Chemotherapeutic drugs, Cancer, Semi-synthetic derivatives, Clinic trials, anti-cancer properties, anti-cancer studies, Toxicity, side effects, and safety”. To collect data for studies confirming the anti-cancer properties of DIM/I3C, we considered controlled experiments that assess the anti-tumor activities of I3C and DIM compounds. The main findings on the biological activity of I3C and DIM were recorded by obtaining the model in which was tested, response, IC_50_ or tested concentration, mechanisms, or molecular target and reference. To collect data for all review studies were excluded according to the following criteria: (i) abstracts, comments, case reports, unpublished data, letters, and reviews. (ii) Research articles without access to the full text or incomplete data. We included original articles in English, peer-review, and the documents’ complete texts were preliminarily examined to select the material.

### General characterization of 3,3′-diindolylmethane (DIM) /Indole-3-carbinol (I3C)

Cruciferous vegetables like cabbage, cauliflower, Brussels sprouts, and broccoli contain in the raw form indole glucosinolates (also named glucobrassicins). These molecules are converted by endogenous enzymatic digestion to several polyaromatic indolic compounds, which could be the reason for many of the physiological effects of these foods; however, among the indoles, only I3C is available at commerce as an off-white solid [10–12].

**Fig. 1:**
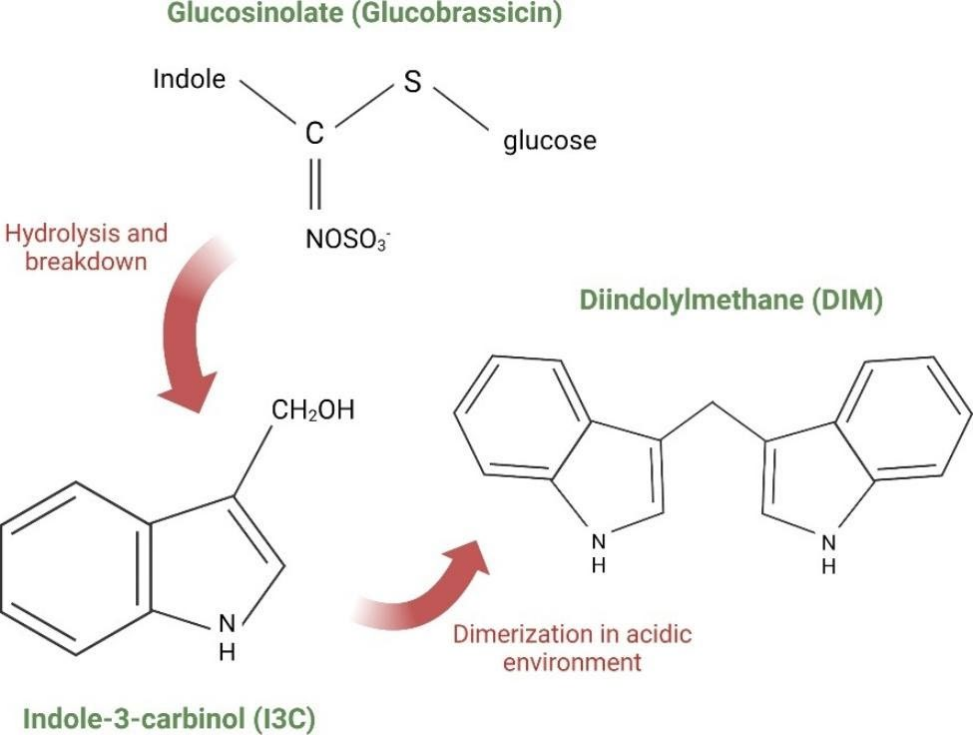
Cleavage of glucosinolate leads to indole-3-carbinol (I3C) formation. Acid condensation products such as 3,3’-diindolymethane are formed due to the acidic environment of the stomach and gut

The synthesis of I3C occurs in sequential steps; first, an intermediate complex is developed after glycolysis from the glucosinolate structure. In the second stage, the sulfur cyanate of the intermediate is separated, and the indole ring of I3C is created [10, 13, 14]. The discarded cyanate makes this molecule different from the aliphatic or aromatic isothiocyanates. Due to the presence of a benzylic system equivalent in the 3-position of indoles, they presented behavior related to this molecule. It is reported that I3C is considered a vinylogous carbinolamine since the presence of an sp^2^ hybridized nitrogen atom (Fig. 1) at one end of the electron-devocalizing triad system making a more stable “benzylic” system, than an all-carbon one [14, 15].

I3C has a molecular weight of 147.17386 g/mol and is stable between 2 and 80 °C; it presents a melting range from 96 to 99 °C and a solubility of 3.75 mg/ml (water). I3C can suffer transformation under the acidic environment of the stomach and the gut into other conjugates (Fig. 1) and is chemically unstable at in vitro cell culture conditions [13, 14]. Among the more recognized of these are indole-3-tryptophan and DIM; the last one is the superior and more bioactive product of I3C oligomerization obtained in a low pH condition and, as I3C, presents protective effects in chemically induced cancer and other diseases; meanwhile, the other I3C conjugate’s functions are yet unknown [12].

Despite all the properties that present I3C and DIM, some limitations restrict their pharmacological application and complicate preserving their efficacy during storage or after oral administration. I3C is photo- and thermo-sensible [16, 17]. Furthermore, the fast condensation of I3C into different oligomeric products, mainly into DIM, in an acidic environment is one of the major challenges in evaluating their individual efficacy [18].

Regarding solubility, I3C has a low water solubility (3.75 to 7 mg/ml), which limits its use as a medical treatment [14, 19]. It has been reported that alternatives to enhance the I3C solubility, such as encapsulation, could improve its release time. Different systems have been developed using materials like biopolymers and lipids [16, 18, 20].

The bioavailability of I3C and DIM is another parameter that could decrease their effectivity and complicate clinical translation. These compounds suffer fast oxidation, metabolization, and elimination, triggering a rapid serum level decreasing. It has been reported that I3C in neutral cell culture media suffered dimerization into DIM at 50% just in 24 h [21].

Different pharmacokinetics studies have been developed to evaluate the activity of the I3C and its condensation products, indicating remarkable results [17, 22, 23]. In this context, it is reported that the I3C is rapidly absorbed and distributed into different tissues and fluids such as the liver, kidney, heart, lung, brain, and plasma after oral administration of 250 mg/kg to mice, detecting the highest concentrations in the liver and kidney. On the other hand, DIM was found in plasma 15 min after the I3C dosage and was still quantifiable for 6 h [17].

In another study, the authors compared the level of DIM in rat plasma by different administration forms, demonstrating that the highest bioavailability was observed in the liquid oil administration compared to crystalline DIM forms [22].

In a prominent pharmacokinetic study of I3C, women received different doses of I3C (from 400 to 1200 mg), and the plasma was analyzed over periods of time. The results indicated that DIM was detectable only before 12 h from the test and that I3C was undetectable in plasma due to its high instability. I3C is promptly absorbed, distributed, and eliminated from plasma and tissues, being undetectable after 1 h of the dose [24].

Due to the instability of I3C under various circumstances, such as acidic conditions, light, or heat, it is challenging to maintain its effectiveness during storage or after oral administration [18].

Besides, the dimerization products partially contributed to their in vivo activity, complicating the elucidation of their individual properties against different diseases; thus, studies of these two molecules have been conducted to analyze their diverse effects [13, 23, 25, 26].

Recently, it has been reported that the in vivo analysis of DIM administration exhibited a reduction in platelet aggregation and reactive oxygen species (ROS) and an antithrombotic effect [11, 26, 27]. Other authors reported that the antithrombotic activity and antiplatelet aggregation of I3C are derived from DIM [28]. In 2022, the I3C oral administration in the middle cerebral artery occluded rats triggered the reduction of neurological deficits, brain infarction by 20%, and brain water content by 75% compared with intravenous administration [28]. Furthermore, the concentration of DIM derived from oral administration of I3C was 5-fold that of the intravenous, suggesting that the DIM presence is indispensable for effective ischemic stroke treatment.

Generally, these molecules reduce oxidative stress, impede DNA synthesis to influence target cells’ activation, proliferation, and apoptosis, and inhibit the proinflammatory cytokines and chemokines from reducing induced liver injuries [29, 30]. Moreover, I3C and/or DIM affect multiple signaling pathways and target molecules controlling cell division, apoptosis, or angiogenesis deregulated in cancer cells [6, 15, 25]. The principal pathways targeted by I3C/DIM and their action mechanisms will be discussed in subsequent sections due to the topic’s relevance.

### Plant sources and metabolism of indole-3-carbinol

Since ancient times, it has been thought that vegetables and their extracts possess therapeutic and curative effects. Nowadays, healthy state people are associated with diets containing high amounts of cruciferous vegetables such as horseradish, collard greens, cabbage, cauliflower, Brussels sprouts, and broccoli [31–33]. Various investigations have suggested that consuming cruciferous vegetables inversely correlates with some cancer types, including kidney, breast, esophagus, colorectal, pharynx, and oral cavity cancers [34–36]. Although cruciferous vegetables are rich in numerous phytochemicals, their potential anti-cancer activity has been mainly attributed to their contents of natural compounds called glucosinolates [37]. However, glucosinolates are not directly responsible for the health benefits of crucifers; these benefits are accredited to phytochemicals resulting from their enzymatic break [38]. The main of these compounds is I3C, produced by the enzymatic hydrolysis of 3-indolylmethyl glucosinolate (also known as glucobrassicin), an indole glucosinolate. The production of I3C is catalyzed by the endogenous enzyme myrosinase, which is kept separately from glucosinolates in different plant compartments [39]. The separation is probably related to the need to produce compounds derived from glucosinolate hydrolysis under certain conditions. For example, it could constitute a mechanism of defense of plants against herbivory animals because glucosinolates breakdown creates thiocyanates, oxazolidine-2-thiones, nitriles, isothiocyanates, and epithionitriles, substances that may result toxic for herbivores [38].

**Fig. 2:**
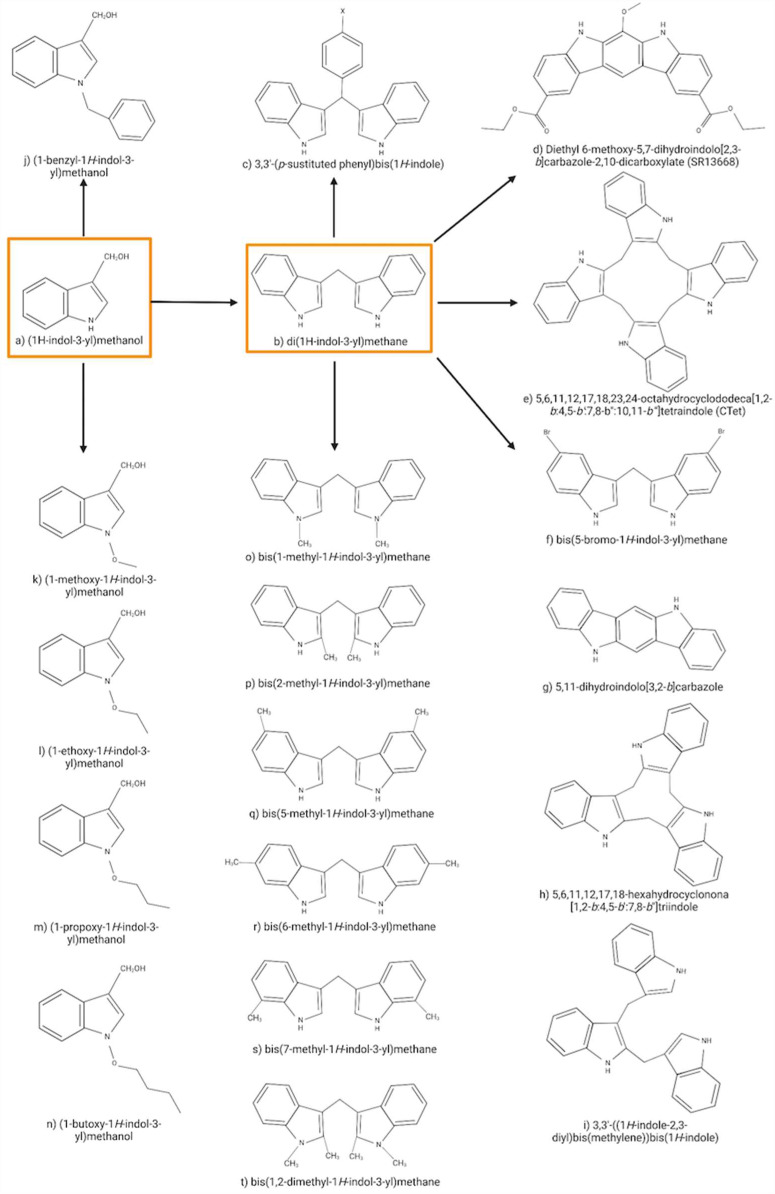
Structure of the main I3C/DIM derivatives related to the anti-cancer activity

Chewing or any mechanical injury (for example, chopping) damages the plant cells, leading to exposure of glucobrassicin to myrosinase. The enzyme leads to the breakdown of glucobrassicin, forming glucose and some unstable intermediates that include thiohydroximate-O-sulfate and 3-indolylmethyl isothiocyanate. Then, these intermediates give rise to I3C and indole-3-acetonitrile (I3N); subsequently, I3C can form conjugates with other plant metabolites (Figs. 1 and 2). In the human stomach, the acidic pH allows I3C to give rise to some condensation products, such as 5,11-dihydroindolo-[3,2-b]carbazole (ICZ) and DIM, which also have biological activities [40].

From a dietary point of view, boiling cruciferous vegetables can alter these enzymatic reactions because it causes the denaturation and degradation of the myrosinase enzyme, preventing the formation of I3C [41]. Thus, intact glucosinolates directly pass to the bowel, where they are processed by intestinal microbiota. Although particular intestinal bacteria exhibit myrosinase activity, production of I3C is on a lesser scale; furthermore, oligomers from I3C are less likely to produce in the alkaline pH of the bowel [42, 43]. Therefore, cooking cruciferous vegetables affects the biological properties of I3C.

In this regard, there is a growing interest in I3C because some studies have pointed out that this phytochemical and its oligomers may modulate the expression and activity of biotransformation enzymes involved in the metabolism and elimination of many biologically active compounds, including carcinogens, drugs, toxins, and steroid hormones. These enzymes include phase I enzymes, such as CYP isoforms, and phase II enzymes, such as glutathione S-transferases (GSTs), NAD(P)H:quinone oxidoreductase 1 (NQO1), and UDP-glucuronosyltransferases (UGTs). I3C can affect the activity of these enzymes in different ways. For example, I3C can induce the expression of some CYP isoforms, such as CYP1A1 and CYP1A2, which are responsible for converting pro-carcinogens into carcinogenic metabolites. However, I3C can also promote the expression of some phase II enzymes, such as GSTs and NQO1, which can detoxify these carcinogenic metabolites and facilitate their excretion. Moreover, I3C can inhibit the activity of some CYP isoforms, such as CYP1B1 and CYP19, which are involved in the biosynthesis of estrogen and other steroid hormones. By reducing estrogen and other steroid hormone levels, I3C may prevent the stimulation of hormone-dependent cancers, such as breast and prostate cancers. Likewise, it has been suggested that I3C possesses beneficial biological activities that include anti-inflammatory and antioxidant properties [44]. Finally, I3C might regulate signaling pathways associated with angiogenesis, apoptosis, cell proliferation, and invasion [45, 46]. These pathways are dysregulated in cancers; thus, I3C may possess chemopreventive and anti-cancer properties.

Consequently, diverse research groups have investigated the plant sources containing the precursor of this phytochemical [47–51]. According to comparative studies, crucifers are vegetables that have more glucosinolates. Although all crucifers contain those bioactive phytochemicals, their content profiles vary among plant species (see Table 1). Notably, glucobrassicin generally represents 8–14% of the total glucosinolates in edible parts; however, in some cases, it can reach up to 80%.

**Table 1:** Contents of total glucosinolates in edible cruciferous vegetables [47–51]

Crucifer	Total glucosinolates (mg/100 g fresh weight)	Most abundant glucosinolates
Broccoli	40.80–127.49	4-(methylsulfinyl)butyl-glucosinolate 4-(methylthio)butyl- glucosinolate 2-hydroxy-3-butenyl- glucosinolate
Brussels sprouts	80.12–445.5	3-indolylmethyl-glucosinolate Allyl-, 3-butenyl-,2-hydroxy-3-butenyl- glucosinolate 3-melhylsulfinylpropyl- glucosinolate
Cauliflower	11.70–78.60	Allyl-, 3-(methylthio)propyl glucosinolate 3-methylsulfinylpropyl- glucosinolate 3-indolylmethyl- glucosinolate
Cabbage	18.79–90	3-indolylmethyl- glucosinolate Allyl- glucosinolate 3-butenyl- glucosinolate
Collars	200.67	Allyl- glucosinolate 3-butenyl- glucosinolate 2-hydroxy-3-butenyl- glucosinolate
Horseradish	160.12	Allyl- glucosinolate 3-indolylmethyl- glucosinolate 3-methylsulfinylpropyl glucosinolate
Kales	62.20–317.11	3-indolylmethyl- glucosinolate 2-hydroxy-3-butenyl- glucosinolate 5-methylsulfinylpentyl- glucosinolate Allyl- glucosinolate
Kohlrabi	19.07–109.30	3-indolylmethyl- glucosinolate 3-(methylthio)propyl- glucosinolate 4-(methylthio)butyl- glucosinolate
Mustard	118.09–544.47	Allyl- glucosinolate 3-butenyl- glucosinolate 2-phenylethyl- glucosinolate
Radish	44.79–172.40	4-methylthio-3-butenyl glucosinolate 3-indolylmethyl- glucosinolate 5-methylsulfinylpentyl- glucosinolate
Turnip	20.44–140.48	Phenethyl- glucosinolate 4-Pentenyl- glucosinolate

### Semi-synthetic derivatives

Due to the anti-cancer effects of I3C and DIM, associated with diverse polyphenolic derivatives formed in the acidic digestive process, these molecules have been proposed as models for anti-cancer agents. Nevertheless, I3C reacts in acidic conditions towards multiple condensation products (Fig. 2) [38, 52], yielding metabolites with unpredictable pharmacokinetic properties, making it difficult to achieve therapeutic concentrations [53]. The metabolites are thus frequently accessed following semi-synthetic approaches.

DIM derivates with broad anti-tumor potency (Fig. 2a-e) include 3,3’-(p-substituted phenyl) bis(1* H*-indole) (1c) as an effective chemotherapeutic against colon cancer, breast cancer, and myelogenous leukemia. The novel agents exhibit a molar potency in inducing apoptosis or cell cycle arrest through signaling pathways (peroxisome proliferator-activated receptor and PPARγ agonists; diethyl 6-methoxy-5,7-dihydroindolo[2,3-b]carbazole-2,10-dicarboxylate (SR13668), Akt inhibitor [54, 55]. The cyclic derivate 5,6,11,12,17,18,23,24-octahydrocyclododeca[1,2-b:4,5-*b’*:7,8-*b’’*:10,11-*b’’’*]tetraindole (CTet) is synthesized from 2,3’-DIM in the presence of formaldehyde under acidic conditions [52], inhibiting cell proliferation through overexpression of p21/CDKN1A in estrogen receptor-positive and triple-negative breast cancer cells [53].

bis(5-bromo-1 H-indol-3-yl)methane (Fig. 2f) is a representative example of symmetrical ring-substituted DIM analogs (C-DIM), which are obtained from the condensation between the corresponding indole derivatives and aliphatic or aromatic aldehydes. The C-DIMs inhibited pancreatic, breast, liver, and uterus cancer cell survival and were up to two times more potent than DIM [56]. Additionally, they have displayed selectivity, being poorly cytotoxic toward healthy cells [57].

**Fig. 3:**
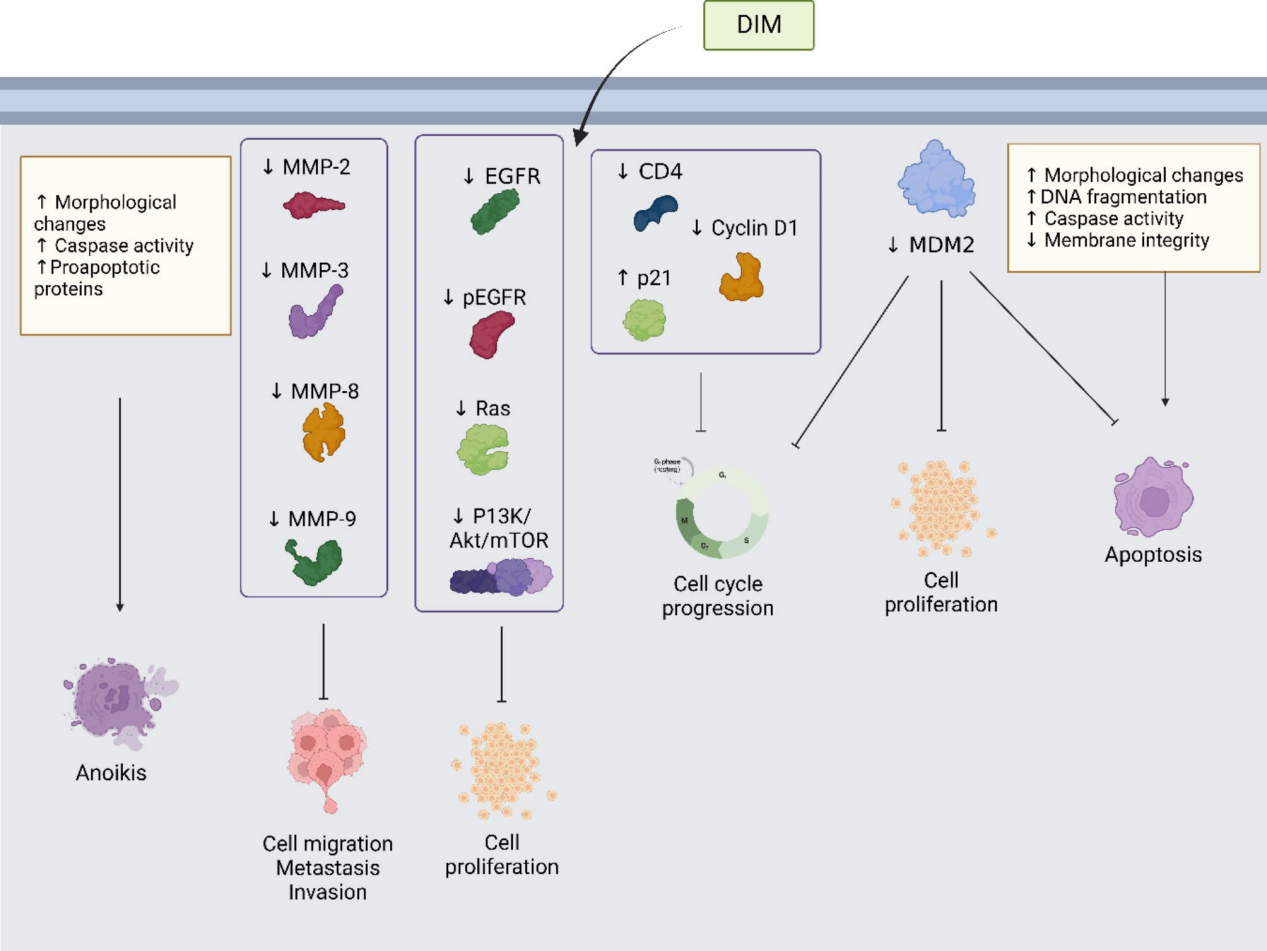
Molecular mechanism of anti-tumoral properties of DIM. The anti-cancer activities of DIM involve the positive (↑) and/or negative (↓) regulation of protein expression and activities, regulating cellular processes considered hallmarks of cancer. For details, see the text

Antiestrogenic, antiandrogenic, and aryl hydrocarbon receptor (AhR) agonist activities have also been identified for condensation products of I3C. They are then leading to a potential hormone therapy aimed at slowing or stopping the growth of hormone-sensitive cancer tumors and avoiding the disruption in AhR expression/activity to result in altered intestinal homeostasis and carcinogenesis [58], with DIM being an androgen receptor antagonist. 5,11-dihydroindolo[3,2-*b*]carbazole (ICZ) (Fig. 2g) and the trimers 5,6,11,12,17,18-hexahydrocyclonone[1,2-b:4,5-b′:7,8-b″]tri-indole (CTr) (Fig. 2h) and 3,3’-((1* H*-indole-2,3-diyl)bis(methylene))bis(1* H*-indole) (Fig. 2i) have shown a remarkable activation of AhR. Additionally, the LTr1 trimer exerts antiandrogenic activities [59], with cell proliferation assays in human MCF-7 cells showing suppression of proliferation of estrogen receptor-negative and positive cells [60], and a recent study informing on a slight agonist effect on the estrogen receptor [11, 59].

The most potent non-condensable derivatives of I3C known are N-alkoxy-I3C and (1-benzyl-1* H*-indol-3-yl)methanol. In vitro, enzymatic assays demonstrated that I3C and, at lower concentrations, (1-benzyl-1* H*-indol-3-yl)methanol (Fig. 2j) act as noncompetitive allosteric inhibitors of elastase activity, with (1-benzyl-1* H*-indol-3-yl)methanol as the most potent I3C derivative known (approximately 1000-fold more potent than I3C) [11, 61].

The antiproliferative response of I3C derivatives containing one- to four-carbon N-alkoxy substitutions (Fig. 2k-n) has been reported, with N-alkoxylation leading to inhibited dehydration and reactive indolenin formation. Compared to I3C, half of the maximum growth (EC50) arrest responses occurred at 23, 50, 217, and 470-fold lower concentrations for N-methoxy-I3C, N-ethoxy-I3C, N-propoxy-I3C, and N-butoxy-I3C, respectively, relating their efficiencies with the length of the alkoxide chain [62, 63]. Other semi-synthetic derivatives of I3C/DIM include the methyl-substituted DIMs (Fig. 2o-t), such as, bis(1-methyl-, bis(2-methyl-, bis(5-methyl-, bis(6-methyl-, and bis(7-methyl-1 H-indol-3-yl)methane [11]. These I3C/DIM derivatives were found to retain and/or enhance their chemoprotective and chemotherapeutic properties, thus representing potentially appealing anti-cancer agents.

### Current medical applications – official treatment or traditional medicine

Functional foods are known for their abundance of active compounds, also known as phytochemicals, which are highly beneficial for promoting good health [26]. Dietary supplements, commonly referred to as nutraceuticals or food supplements, offer a concentrated source of bioactive agents found in food that is presented in a non-food form. The supplements are taken in dosages that surpass what could be obtained from regular nutrition and are intended to improve overall health [64]. The use of dietary compounds in chemoprevention has gained significant attention as a feasible method for preventing cancer. This is based on epidemiological studies that have established a strong link between consuming plant-based foods rich in phytochemicals, such as whole grains, vegetables, and fruits, and a lower incidence of cancer [65].

Humans are primarily exposed to I3C by consuming Brassica vegetables, commonly included in the diets of both Eastern and Western cultures [26, 66]. In the United States population, the daily intake of I3C from cruciferous vegetables has been estimated to be less than 2.6 mg. In the United Kingdom, the estimated intake is 0.1 mg/kg body weight; in the Japanese diet, it is 1.6 mg/kg for an individual weighing 70 kg [67, 68].

Clinical trials investigating the therapeutic potential of I3C have used a daily dose of 200 to 400 mg, which is also the recommended dosage mentioned on the packaging of I3C products. For a person weighing 70 kg, the daily doses correspond to exposures of 2.9 to 5.7 mg/kg [69]. Table 2 provides an overview of completed cancer-related clinical trials that have assessed I3C, DIM, and Brassica vegetables in their respective phases. Both I3C and DIM have been identified as effective chemopreventive agents [70]. I3C is an FDA-approved nutritional supplement widely advertised and marketed as a dietary supplement, available for purchase at health food stores, pharmacies, and online. It may be sold as a single-ingredient product or as a combination nutraceutical containing various botanicals and/or vitamins [69].

**Table 2:** Examples of completed clinical trials related to cancer [77]

Tested compound	Condition	Clinical Trial number	Dosage	Objective	Outcomes
I3C	Cancer	Clinical Trial-Phase 1- NCT00100958	---	To study the side effects and best dose of I3C and to see how well it works compared to a placebo in preventing cancer in healthy participants.	No study results have been posted.
I3C	Breast Cancer	Clinical Trial-Phase 1- NCT00033345	400 mg daily for 4 weeks, followed by a 4-week period of 800 mg daily	To study the effectiveness of I3C in preventing breast cancer in nonsmoking women at high risk for breast cancer.	No study results have been posted.
DIM Supplement	Breast Cancer	Clinical Trial-Phase 3- NCT02525159	75 mg for 30 days	To evaluate the effectiveness of supplementation with DIM to increase the urinary ratio of estrogen metabolites 2 hydroxyestrone:16 alpha-hydroxyestrone in premenopausal women at risk of breast cancer.	No study results have been posted.
Microencapsulated DIM (BioResponse-DIM)	Prostate Cancer	Clinical Trial-Phase 2- NCT00888654	225 mg twice daily for 14–72 days	To study how well DIM works in treating patients with stage I or stage II prostate cancer undergoing radical prostatectomy.	-A mean level of 14.2 ng/g of DIM was found in prostate tissue after treatment. -The serum levels of PSA, testosterone, and DIM pre- and post-treatment were in ng/ml 6.4 and 5.8, 301 and 388 and 0.0 and 7.5, respectively. -The levels of androgen receptor in prostate tissue (units: intensity x % cells stained) were 278 in pre-treatment and 245 and post-treatment. -Two people out of 41 enrolled, presented headache, reported as a severe adverse event.
Microencapsulated DIM (BioResponse-DIM)	Cervical Dysplasia	Clinical Trial-Phase 3- NCT00212381	2 mg/kg/day	To determine if oral DIM, is associated with the regression of cervical dysplasia in otherwise healthy women.	No study results have been posted.
Brassica vegetables	Prostate Cancer	Clinical Trial - NCT00607932	At least 2 servings (½ cup/serving) daily for 6 months	To study the side effects and how well Brassica vegetable work compared with I3C in treating patients with PSA recurrence after surgery for prostate cancer.	No study results have been posted.

Dietary supplements marketed as compounds are subject to regulation under the Dietary Supplement Health and Education Act of 1994. However, I3C is not listed as a Generally Recognized Safe substance by the FDA [69].

On the other hand, the European Commission considers I3C and DIM as novel foods in its EU Novel food catalog. However, a safety assessment under Novel Food Regulations is required before they may be placed on the market in the EU as a food or food ingredient [71].

I3C-containing supplements are promoted for their potential health benefits, which include cancer prevention, antioxidant protection, hormone regulation, immune system support, and detoxification of the liver and intestines [72]. As mentioned, DIM is a metabolite of I3C; subsequently, the supplements we find on the market are formulated from I3C and/or DIM. Some commercial products, generally presented in capsule-type dosage form, are Genius Estrogen Balance-DIM, Indonal partner for the woman; Indonal Man; Zazzee Naturals-DIM, Gynmax; Nutricost-DIM, ProstaIN; INDOL-IN, Now Foods-I3C and GRAV-IN. The products contain, as declared, between 150 and 400 mg of the active substance [64], quantified by high-performance liquid chromatography method using core–shell column for separation of I3C and its condensation/degradation products an amount of 0.0 mg of I3C in INDOL-IN and GRAV-IN, and amounts much lower than what was declared in Indonal Man and ProstIN. This evidence generates a critical need to regulate this type of supplement that guarantees the daily consumption of the active substance by people who seek a benefit associated with I3C and DIM.

Studies have shown that taking pure I3C as a dietary supplement, equivalent to consuming one-third of a head of cabbage daily, can reverse precancerous changes in women with stage II and III cervical dysplasia. A diet high in cruciferous vegetables or I3C supplements can cause tumor regression or reduce the rate of growth or recurrence in two-thirds of patients with recurrent laryngeal papillomatosis [73]. Research has indicated that dietary indoles, found in Brassica plants, may offer potential protection against hormone-dependent cancers [74]. Oral administration of I3C was found to be a potential preventive agent against breast cancer by impacting human estrogen metabolism in 1990 [75]. DIM has demonstrated the ability to affect estrogen metabolism in women and is the only detectable analyte in plasma following administration of I3C [74]. In a separate human study, participants were given a daily dose of 500 mg of I3C for one week, significantly increasing estradiol 2-hydroxylation from 29.3 to 45.6%. These findings suggest that I3C strongly impacts estradiol metabolism in humans, offering a potential new approach to chemoprevention against estrogen-dependent diseases [75].

In a pilot study examining the impact of absorbable DIM supplements (BioResponse-DIM®) on urinary hormone metabolites in postmenopausal women aged 50–70 with a history of early-stage breast cancer, the treatment group received a daily dose of 108 mg DIM for 30 days while the control group received a daily placebo for 30 days. Results showed that the DIM-treated group had significantly increased levels of 2-hydroxy estrone, DIM, and cortisol compared to the placebo group. Furthermore, DIM was found to increase the 2-hydroxylation of urinary estrogen metabolites [74].

Recurrent respiratory papillomatosis (RRP) is a viral condition caused by HPV that affects the vocal cords and airways. In a trial to evaluate the potential benefits of I3C in RRP treatment, 33 patients were administered an I3C nutritional supplement (200 mg PO BID for up to 4 years and 8 months). I3C modulates estradiol metabolism by inhibiting the production of 16α-hydroxy estrone, a genotoxic and tumor-promoting metabolite that causes inappropriate DNA synthesis. On the other hand, I3C promotes the production of 2-hydroxy estrone, a metabolite effective against hormone-dependent cancers, and inhibits papilloma growth. In patients who did not respond to I3C, either the compound could not significantly alter estrogen metabolism, or the disease was caused by an unknown serovar of HPV [72, 76].

### Studies confirmed the anti-cancer properties of 3,3´-diindolylmethane (DIM) /Indole-3-carbinol (I3C)

As previously mentioned, the anti-tumoral activities of DIM and I3C are based on mechanisms involving NF-κB, Akt, Wnt, PI3K/Akt/mTOR, and AhR signaling. However, miRNAs that function as tumor suppressors are upregulated by DIM [78]. Nevertheless, pre-clinical studies to support the chemotherapy activity of I3C and DIM are limited.

Since xenobiotic substances cause adverse effects and considering that I3C activates multiple signaling pathways, the toxicology of I3C is an important issue in determining at what concentration this compound triggers or accelerates side effects. In clinical trials, 200 to 400 mg doses are used to evaluate the therapeutic potential of I3C, corresponding to 2.9 to 5.7 mg/kg for a 70 kg person. Previous reports indicate that I3C causes reversible toxicity to the gastrointestinal tract in an immune-compromised rodent model [79], suggesting that the intestine is the main target organ for the secondary side effects of administering this phytochemical compound. According to the single ascending dose report, the maximum tolerated single dose of I3C is 400 mg, and doses ≥ 600 mg cause gastrointestinal discomfort due to contaminants such as 3-methylindole. However, in the absence of contamination, the multiple-dose study using 400 mg twice daily had no side effects, suggesting that this dose of I3C is well tolerated [24]. In addition, for male and female mice, the lethal doses 50% (LD_50_) of I3C are 444.5 mg/kg and 375 mg/kg for the intraperitoneal route, respectively, whereas, for intragastric administration, LD_50_ are 1410 and 1759 mg/kg for male and female mice, respectively [80]. Furthermore, I3C doses below LD_50_ will not cause damage to the mice organism. No toxic effects were observed in mice in intraperitoneal injections administered at 250 mg/kg or 550 mg/kg intragastrically [80].

DIM (2.5, 5, or 10 mg/kg body weight) administered intraperitoneally or orally inhibited rat mammary tumor growth [81] and induced apoptosis and cell proliferation arrest, resulting in decreased tumor growth in TRAMP-C2 mouse prostate cancer model [82].

In addition, oral DIM administration (10 mg/kg body weight/d) reduced metastasis in an in vivo lung model (4TI murine mammary carcinoma cells injected into syngeneic female BALB/c mice) [83]. In the K14-HPV16 transgenic mouse model, DIM inhibited the development of cervical lesions [84], and 1000 ppm of DIM increased serum interferon-gamma levels (IFN-γ) in the K14-HPV16 transgenic mouse model, suggesting that it is the minimum effective dose of DIM [85].

Synthetic DIM derivatives regulated cellular processes associated with hallmarks of cancer. Then, DIM-1 and DIM-4 induce apoptosis and anoikis but also have anti-migratory effects in breast cancer cells [86]. Further, DIM analogs (C-DIMs) including 1,1-bis(3′-indolyl)-1-(p-methoxyphenyl) methane (DIM-C-pPhOCH3) and 1,1-bis(3′-indolyl)-1-(p-hydroxyphenyl) methane (DIM-C-pPhOH) function as a NR4A1 activator or deactivator, respectively. C-DIMs induce cell death via NR4A1-dependent and -independent pathways [87].

Also, I3C and DIM present pro-apoptotic and antiproliferative effects but inhibit cell growth in vitro and in vivo, as described in Table 3.

**Table 3:** Anti-tumor activities of I3C and DIM compounds

Compound	Model	Response	IC_50_ or tested concentration	Mechanisms or molecular target	Reference
I3C	Mouse model of colitis-associated colorectal tumorigenesis (AhR +/+)	Decreased colitis-associated tumor	ND	AhR has a protective role in colitis-associated colorectal tumorigenesis.	[92]
I3C/DIM	Apc^Min/+^ mice	Suppresses intestinal carcinogenesis	ND	AhR	[93]
DIM	Prostate cancer	Up regulate the expression of miRNAs (let-7, miR-34a, miR, and 150-5p) miR-92a	ND	Targets of those miRNAs are EZH2 Notch-1, AR, Ahr and RANKL	[78]
	H295R human adrenocortical carcinoma cell	Induce cytochrome P450 1A1, 1B1 and 19	ND	Induced ethoxyresorufin-O-deethylase (EROD) activity and aromatase activity	[94]
	Postmenopausal American women aged 50–70 year with a history of early-stage breast cancer	DIM increased the 2-hydroxylation of estrogen urinary metabolites	Tested concentration: 108 mg DIM/day for 30 days.	DIM-treated subjects showed increased levels of 2-hydroxyestrone (2-OHE1) and cortisol.	[74]
	64 patients with biopsy-proven cervical intraepithelial neoplasia (CIN) 2 or 3	High rate of improvement in lesion number.	Oral DIM at 2 mg/kg/day for 12 days.	Improvement in confirmed CIN 2 or 3 lesions according to Pap smear, HPV, colposcopy, biopsy, and physical examination.	[95]
DIM-1 and DIM-4,	Cancer cell lines	Induce apoptosis and anoikis	ND	Compounds induce morphological analysis, nuclear fragmentation, membrane integrity assay, caspase activity measurements, and modulation of pro/anti-apoptotic proteins.	[86].
2,2’-Diphenyl-3,3’-diindolylmethane (DPDIM)	Triple-negative breast cancer	Induces apoptosis in vitro in breast cancer cells (MCF7, MDA-MB 231, and MDA-MB 468) and in vivo in 7,12-dimethylbenz[α]anthracene (DMBA) induced Sprague-Dawley (SD) rat mammary tumor	IC_50_ ca. 10 µmol/L; triple-negative refers to breast tumor cells lacking ER/estrogen receptor and PR/progesterone receptor, and no HER-2 overexpression	Negatively regulates the activity of EGFR and its downstream molecules like STAT3, AKT, and ERK1/2	[96]
4,4′-Dibromo-, 4,4′-dichloro-, 7,7′-dibromo-, and 7,7′-dichloro DIM	Prostate cancer cells (LNCaP cells)	Inhibits DHT-stimulated growth of LNCaP cells. Induced autophagy	Tested concentrations: 10 and 30 µM and 0.3–30 µM	Suppressed androgen receptor expression and induced apoptosis and necrosis by activating caspases-3, -8, and − 9, and induced expression of Fas, FasL, DR4, and DR5. Induced autophagy in prostate cancer cells by activation of AMP-activated kinase (AMPK) signaling and astrocyte-elevated gene 1 (AEG-1)	[97–99]
1,1-Bis(3′-indolyl)-1-(*p*-substituted phenyl)methanes	Colon cancer cells (SW480 cells)	Inhibits the growth of SW480 tumors in vivo. Induce apoptosis in colon cancer cells.	Tested concentrations: 2.5 to 7.5 µmol/L	Induce peroxisome proliferator-activated receptor γ (PPARγ). Induce apoptosis in colon cancer cells and tumors by enhancing JNK phosphorylation that appears to be independent of activation of classical markers of endoplasmic reticulum stress	[100, 101]
1,1-Bis(3′indolyl)-1-(substituted aromatic)methanes (i.e. C-DIMs)	Breast cancer cells (MDA-MB-231) and pancreas cancer cells (BxPC-3), tumor cell lines 518A2 melanoma, KB-V1/Vbl cervix carcinoma and HT-29 colon carcinoma	Growth inhibition Apoptosis	IC_50_ < 5 µmol/L for BxPC-3 IC_50_ = 1.0 µmol/L for 518A2 IC_50_ = 3.0 µmol/L for KB-V1/Vbl, IC_50_ = 6.3 µmol/L for HT-29	DIM activates or inactivates multiple nuclear receptors, induces endoplasmic reticulum stress, decreases mitochondrial membrane potential, and modulates multiple signaling pathways, including kinases	[56]
1,1-Bis(3′-indolyl)-1-(4-pyridyl)-methane (DIM-C-Pyr-4)	Breast cancer cells (MCF-7 and ZR-75)	Contradictory results. DIM-C-Pyr-4 interacts with Chicken ovalbumin upstream promoter-transcription factor I (COUP-TFI), suppressing estrogen-induced gene expression while enhancing the motility and invasiveness of MCF-7 cells.	ND	Interacts with Chicken ovalbumin upstream promoter-transcription factor I (COUP-TFI) and activated COUP-TFI-dependent early growth response 1 (Egr-1) expression	[55, 102, 103]
1,1-Bis(3′-indolyl)-1-(p-methoxyphenyl)-methane (DIM-C-pPhOCH_3_)	Colon cancer cells (SW480 cells)	Inhibits tumor growth	ND	Activates extrinsic apoptosis pathway and activates Nur77-independent apoptosis.	[104]
1,1-Bis(3′-indolyl)-1-(*p*-methoxyphenyl)-methane (DIM-C-pPhOCH_3_)	Pancreatic tumors in mice and pancreatic cells (L3.6pL)	Inhibits cell and tumor growth and induces apoptosis	Tested concentrations in mice: 25 mg/kg/day	DIM-C-pPhOCH_3_ induced Fas ligand and tumor necrosis factor-related apoptosis-inducing ligand (TRAIL), and induction of TRAIL was dependent on activating transcription factor 3 (ATF3).	[105]
1,1-Bis(3′-indolyl)-1-(p-hydroxyphenyl)-methane (DIM-C-pPhOH)	Breast cancer cells (MDA-MB-231 and SKBR3) and mouse xenograft model	Tumor growth inhibition. Inhibits migration and induces apoptosis	Tested concentrations in mouse: 2 mg/kg/d	DIM-C-pPhOH is an antagonist of nuclear receptor 4A1 (NR4A1) and inhibits NR4A1-regulated pro-oncogenic pathways/genes in breast cancer cells	[106]
1,1-Bis(3′-indolyl)-1-(4-chlorophenyl)-methane (DIM-C-pPhCl)	Pancreatic cancer cells	Inhibits migration and induces apoptosis	ND	DIM-C-pPhCl selectively activated NR4A2 (Nurr1) and had only marginal effects on NR4A1 and NR4A3 activity	[107]
DIM-C-pPhtBu	Pancreatic cancer cells	Induce apoptosis	ND	Induced ER stress included CHOP-dependent induction of death receptor DR5 and subsequent cleavage of caspase 8, caspase 3, Bid, and PARP.	[108]
1,1-Bis(3′-indolyl)-1-(p-chlorophenyl)-methane (DIM-D)	Caco-2 cells	ND	ND	Reduce permeation across caco-2 monolayer	[109]
Bis(triethylammonium) tris[1,1-bis(indol-3-yl)-1-(3,4-catecholate)-methane]vanadate(IV) complex.	Cancer cell lines such as 518A2 melanoma, HCT-116 colon carcinoma (both p53-wildtype and p53-negative cells), triple-negative MDA-MB-231 breast cancer, and Panc-1 and BxPC-3 pancreas cancer cells	Cell cycle arrest	IC_50_ = 1.8-3.0 µmol/L for 518A2 melanoma cells	Inhibition of tumor cell growth led to increased ROS formation and to a decrease of the mitochondrial membrane potential that caused mitochondrial damage, produced reactive oxygen species (ROS), and led to G2/M cell cycle arrest in 518A2 melanoma cells	[110]
Phemindole [3,3′-(4-hydroxyphenylmethylene)-bis-(7-methy-1 H-indole)]	Triple-negative breast cancer cells (TNBC, MDAMB-231).	Apoptosis Cell migration arrest	IC_50_ = 10.9 µmol/L for MDA-MB-231	Phemindole caused mitochondrial-based apoptosis and ROS formation, and ER stress and mediated Store Operated Calcium Entry (SOCE) retardation favored the inactivation of STIM1.	[111]
N-glycosylated DIM derivative (Phemindole)	A549 (non-small cell lung carcinoma), HeLa (cervical cancer cell line), and MCF-7 (breast cancer).	Apoptosis Inhibited migration Arrested cell cycle	IC_50_ = 1.3 µmol/L for A549 lung, IC_50_ = 0.3 µmol/L for HeLa cervix, and IC_50_ = 0.9 µmol/L for MCF-7 breast cancer cells	N-glycosylated DIM derivatives induce apoptosis by upregulation of pro-apoptotic Par-4 (prostate apoptosis response 4) accompanied by suppression of Bcl-2 and GRP78 (glucose-regulated protein 78 kDa) and arrested the cell cycle in the G1 phase	[112]
5,50-Dibromo DIM	Colon cancer cells	Inhibits tumor growth	Tested concentration 30 mg/kd/d	Induces Krüppel-like factor 4 and p21	[113]
	Breast cancer	Apoptosis		Induces caspase-dependent apoptosis, damage to mitochondrial-dependent apoptosis	[114]

ND, No data

The IC_50_ value between DIM and I3C differs by an order magnitude of 10-fold. Thus, DIM has IC_50_ values of 32.1 µmol/L and 25.2 µmol/L against breast cancer cells (MDA-MB-231) and pancreatic cancer cells (BxPC-3), respectively. Furthermore, its IC_50_ > 100 µmol/L against DIM-resistance tumor cell lines melanoma 518A2, vinblastine-resistant cells (KB-V1/Vb1) and colon carcinoma (HT-26). Moreover, DIM derivatives showed differential action depending on the cancer type [88]. For example, the IC_50_ value of pentafluorophenyl derivatives is around 6.2–13.1 µmol/L for androgen-dependent (LNCaP) and androgen-independent (C4-2B, PC-3) prostate cancer lines and in DIM-resistance cell lines such as melanoma, cervical carcinoma (KB-V1/Vbl), and colon carcinoma cells (HT-29) the IC_50_ is around 9.6–16.5 µmol/L [89]. In addition, I3C at 10 µM decrease proliferation in several cancer cell lines, such as cervical cancer cell lines (HeLa, MCF7, and MDA-MB-231) and adenocarcinoma cells (HCT-8). However, the hepatocellular carcinoma cell line (HepG2) showed sensitivity to 5 µM of I3C [90]. Then, these results suggested that DIM and I3C has different mechanism of action depending at least on the type of cancer, the genotype of the cancer cell line and that several factors needed to be addressed to determine whether I3C and DIM have a different effect depending on the type of cancer. For example, both compounds affect cell cycle progression in breast cancer cells and inhibit growth and migration. However, DIM causes apoptosis and anoikis in breast cancer cells, whereas I3C only induces apoptosis.

Furthermore, current clinical trials regarding I3C are mainly focused on breast, prostate, and colon cancers, whereas DIM clinical trials are for cervical dysplasia and breast and prostate cancers. In a phase I dose-escalation study, DIM was orally administered in non-metastatic prostate cancer patients, revealing that PSA levels diminished, and emotional functioning improved over time. Furthermore, twice daily, 225 mg of DIM was recommended for phase II studies [91].

### Mechanism of anti-tumor action of 3,3´-Diindolylmethane (DIM) /Indole-3-carbinol (I3C)

Recently, interest in I3C and its metabolic products, such as DIM, has arisen due to its antiproliferative and anti-inflammatory activities in several types of cancers, such as oral, prostate, breast, colorectal, pancreatic, liver, and gastric cancer (Fig. 3) [115–119].

The first step in the mechanism of action of phytochemical compounds is entry into the cells. Thus, DIM crosses the cell membrane by passive uptake in human breast cancer cell lines [120], suggesting that simple diffusion is the route for absorption.

In esophageal squamous carcinoma and gastric cancer, CDK4 and Cyclin D1 are reduced. At the same time, levels of the cyclin-dependent kinase inhibitor, p21, were increased in the presence of DIM, resulting in G1-phase cell cycle arrest [121, 122].

Furthermore, DIM inhibited mouse double minute 2 homolog (MDM2) in colorectal cancer cell lines, inhibiting cell proliferation and inducing cell cycle arrest and apoptosis [123]. The expression of matrix metalloproteinases, such as MMP2, MMP3, MMP8, and MMP9, are inhibited by DIM, leading to migration and invasion arrest [124].

In triple-negative breast cancer cells, DIM-1 and DIM-4 induce morphological changes, nuclear fragmentation, membrane integrity loss, and caspase activity and modulate pro/anti-apoptotic proteins resulting in apoptosis and anoikis. Besides, DIM-1 and DIM-4 inhibit MMP-2 and MMP-9, leading a cell migration and metastasis arrest. Further, the expression of Ras, phosphorylated forms of PI3K, Akt, and mTOR, and the expression of EGFR and pEGFR are reduced in the presence of DIM-1 and DIM-4, leading to cell proliferation and survival arrest [86].

In addition, DIM arrest the cell cycle by activation or expression of cyclins (Cyclin A, Cyclin B, Cyclin D, and Cyclin E), cyclin-dependent kinases (CDK-1, CDK-2, CDK-4,6), and CDK inhibitors (CDKIs) [125].

DIM inhibits vascular endothelial growth factor (VEGF) activity and Ras signaling induced by VEGF, resulting in anti-angiogenic [125, 126]. Natural targets of DIM include NFκB, RANKL, P13K/Akt, PUMA, Wnt, MTA2, EZH2, Ahr, PTEN, Notch-1, IRAK1, p27, p57, Cdc25A, EGFR, ZEB-1, FoxM1 and Sox4 [78]. All of these targets are suppressed or inhibited by DIM, while E-cadherin and tumor suppressor miRNAs are activated in the presence of DIM [78]. B-DIM down-regulates urokinase-type plasminogen activator (uPA)-uPAR, which decreases VEGF/MMP-9 levels and leads to cell growth arrest and migration inhibition [125].

Evidence suggests that the antiproliferative effect of I3C on breast cancer cells involves estrogen receptor ER-independent and ER-dependent pathways. In the first one, I3C inhibited CDK6 expression and increased CKI (CDK inhibitor) [127], resulting in the G1 block in cell cycle progression. Moreover, I3C reduces the BCL-2 protein levels increasing apoptosis, probably due to a breakdown of mitochondrial membrane integrity [73].

On the other side, in the ER-dependent pathway participates an I3C hydrolysis product (ICZ) that binds to the AhR, which activates the cytochrome P4501A1 through CYP1A1-dependent monooxygenase resulting in estrogen-stimulation growth inhibition [127].

In the ER-dependent pathway, estrogen modulates gene expression causing a rapid response. I3C is involved in triggering the ER-dependent gene expression, such as the upregulation of the expression of CYP1A1 or the ubiquitination of ERα through AhR and AhR/Rbx1, respectively, resulting in antitumor activity. In the canonical AhR signaling, the complex AhR-ARNT (AhR nuclear translocator protein) that is activated by ligands of AhR, interacts with unliganded ER promoting the transcriptional regulation of estrogen response elements [128]. AhR interacts with inhibitory pentanucleotide (GCGTG) dioxin-responsive elements (iDREs), leading to the disruption of the complex between DNA elements required for ER action and the basal transcription machinery [129]. In addition, there are competitive interactions of AhR and response elements for the ER receptor for binding the same region in the promoter, causing AhR-ERα inhibitory crosstalk [130], suggesting that depending on whether ERα is present or not when AhR is involved, it may or may not bind to certain response elements. Since AhR is a modulator of hormone receptor function, androgen and estrogen receptors are sensitive targets to AhR inhibition in a ligand-dependent manner [131]. Complex mechanisms are proposed to explain the anti-estrogenic nature of AhR in breast and other cancers [132, 133] that are associated with the direct interaction with ER, resulting in the inhibition of DNA binding and disruption of coactivator/repressor recruitment [134, 135], that are associated with the anti-proliferative and pro-apoptotic effects of AhR agonist [136]. AhR agonists stimulate CYP1A1/B1-mediated estrogen depletion [137]. AhR acts as a component of the ubiquitin ligase complex, enhancing proteasomal degradation [134, 138].

The 2,3,7,8-tetrachlorodibenzo-p-dioxin (TCDD) activates the AhR by the ERα signaling pathway, and the regulation of estrogen homeostasis [139], and its E2-dependent carcinogenesis mechanism is associated with the AhR/ER crosstalk [140]. However, TDCC is an AhR agonist ligand, but unlike I3C, TCDD is a synthetic and toxic compound.

Considering that AhR/ER crosstalk established for estrogen receptor-positive (ER+) cells and that AhR agonist might be used as a cancer therapeutics opens new possibilities to the medical need for therapy for estrogen receptor-negative (ER^‒^) breast cancers [141].

Since I3C is a ligand of the AhR, there is I3C-dependent activation of the mechanisms involved in the anti-cancer activity of I3C. One of these involved disrupting an ERα/GATA3 cross-regulatory feedback loop due to I3C inducing the ubiquitination and proteasome-mediated degradation of Erα, resulting in the estrogen-dependent growth arrest (cell cycle arrest) [142]. Another example involves the AhR binds to Xenobiotic Responsive Elements (XREs), resulting in a UBE2L3 expression that ubiquitinates E7 leading to the arrest of cell proliferation [143].

Furthermore, I3C increases the expression of PTEN, an important tumor suppressor that inhibits cell proliferation and promotes tumor cell apoptosis suggesting that I3C might inhibit the development of cervical cancer [144]. Moreover, I3C is responsible for the dose-dependent increase of E-cadherin and PTEN in breast cancer cell lines. In detail, PTEN interacts with focal adhesion kinase (FAK), resulting in its tyrosine phosphorylation leading to the inhibition of tumor invasion and metastasis [145]. E-cadherin is a regulator of cell-cell adhesion, and its decreased expression correlates with the progression of breast cancer by increasing proliferation, invasion, and distant metastasis [146, 147].

**Fig. 4:**
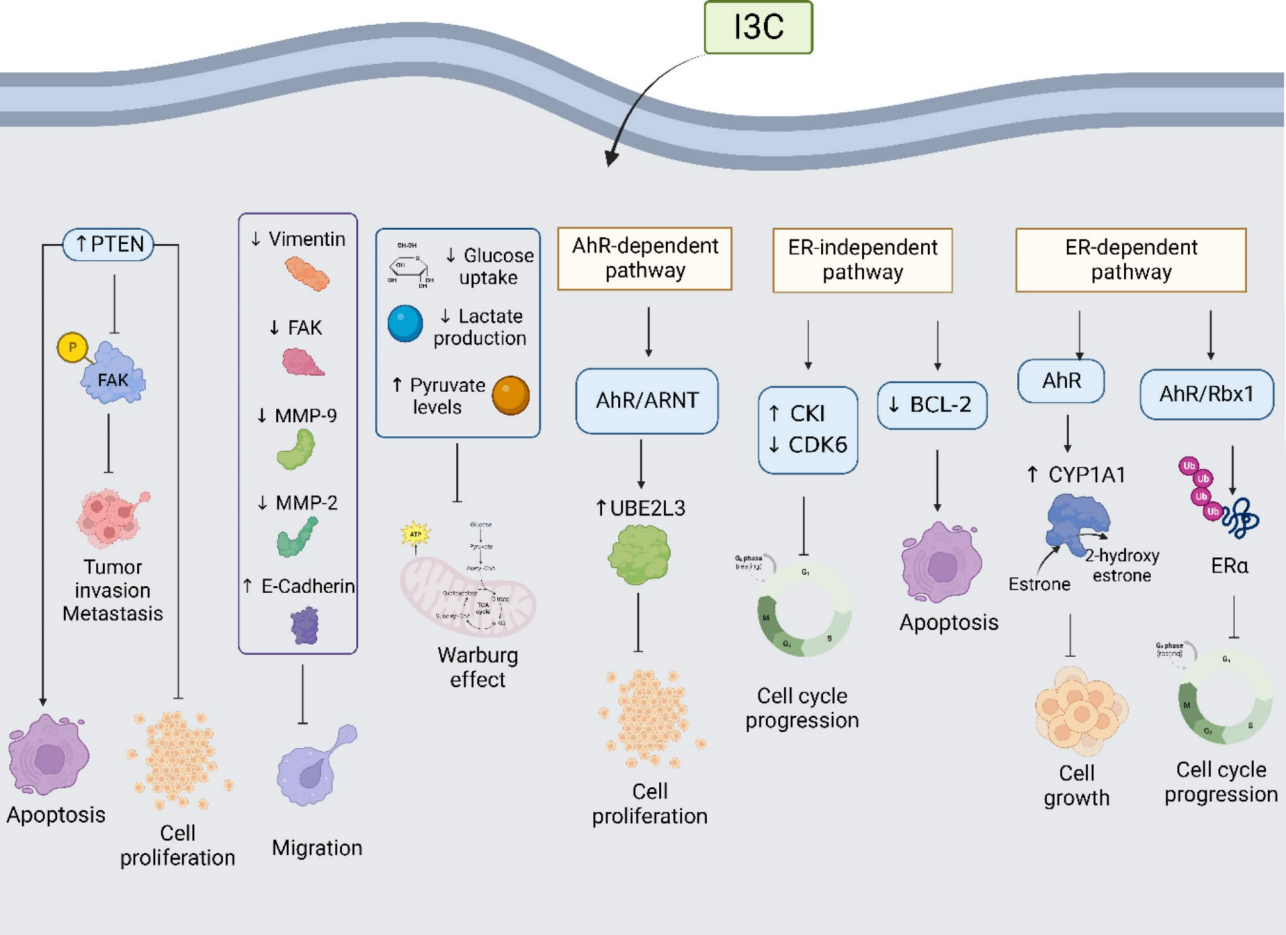
Molecular mechanism of anti-tumoral properties of I3C. The anti-cancer activities of I3C involve the positive (↑) and/or negative (↓) regulation of protein expression and activities, regulating cellular processes considered hallmarks of cancer. For details, see the text

In breast cancer cells, I3C and ICZ treatments decreased vimentin and FAK levels, reduced the activity of MMP2 and MMP9, and increased the E-cadherin levels, resulting in an inhibition of migration through the epithelial-to-mesenchymal transition (EMT) process [148]. Finally, I3C decreases glucose uptake and lactate production and increases pyruvate levels in cervical cancer cells. The metabolic alterations caused by I3C modulates the Warburg effect [149], the mechanisms are summarized in Fig. 4.

### Potential biotechnological studies on in vitro cultures

Many published studies on the anti-cancer properties of DIM address evaluations in simple 2D screens of proliferation, invasion, and cytotoxicity [89, 123, 150–156]. However, due to their lack of representativeness with the tumor microenvironment [157] and their low correlation with in vivo studies [158], 3D cellular models have become more relevant.

It has been noted that chemopreventive effects in premalignant organoid-based models may increase the predictive value in vivo, as presented in the comparative study with the select clinical trial in 2019 [159].

Other approaches to cancer prevention and inhibition include the relevance of using 3D peptidic hydrogel scaffolds due to the effect on bioactive compounds during the digestive process [158]. On the other hand, the phenotypic approach may allow modeling the cell transformation process to identify compounds that have the potential to prevent or attenuate the transformation stimulated by tumor promoters through a 3D model implemented with high-throughput screening [160].

Some studies demonstrate the increase in cellular resistance in spheroids [86, 161] concerning 2D cultures associated with the drug’s mode of action [162]. For this reason, it is possible to consider these 3D models as adequate tools to evaluate chemoresistance since they allow better replication of the cellular behavior of the disease by improving the simulation of the microenvironmental and structural conditions and interactions of the tumor.

On the other hand, epigenetic alteration is crucial in chemotherapy resistance [163]. On this basis, Nikulin et al. [164], using organoids, demonstrated microRNA silencing by DIM suppression of mir-21-5p expression, recapitulating the pharmacological response in vivo.

The microfluidic system has proven to be a relevant platform for investigating the impact of hyperosmotic stress on migration, proliferation, and ion channel/transporter expression changes in metastatic cell lines [165].

Furthermore, given the difficulty of recapitulating the complexity of tumor initiation and progression in vivo, the size- and stiffness-controlled alginate hydrogel microsphere system developed by Rios de la Rosa et al. [166] becomes relevant. The authors demonstrated the similarity between the biomarker expression pattern of the model with the early stage of colorectal cancer.

To better mimic the native tumor microenvironment, 3D cultures can employ extracellular matrix materials [167], hydrogels, microfiber scaffolds, tissue engineering [168], co-cultures [158, 166, 167, 169] and can even make use of 3D bioprinting [170].

It is worth mentioning that 3D models present their challenges. The models tend to be more expensive and can have difficulties in their construction, replication of the microenvironment, and automation of fluid handling for high-throughput screening, and imaging can be burdensome when large scaffolds are used [168].

A suggested strategy is to use 2D cultures for rapid initial screening to identify relevant effects, and 3D models can be helpful for further studies of efficacy or cytotoxicity before using animal models [166].

Additionally, the development of standardized protocols to generate reproducible and reliable results should be considered [167, 171] if employing 3D cell culture systems or combined assay strategies for studying the anti-cancer properties of DIM.

### Toxicity, side effects, and safety

Most studies evaluate DIM’s toxicity, side effects, and safety, and IC3 is adult-oriented [172, 173]. In a study with young rats as a model of the pediatric population, where evaluate the safe oral DIM, a dose of 2.0 (therapeutic dose) or 20.0 mg/kg/day (dose 10x what therapeutic) and whether DIM posed a higher risk in juvenile rats than adults. The authors found no difference in the chemical composition of rat serum or histological changes in the liver, kidney, or bone density. Therefore, DIM does not produce side effects in any group [174].

Female and male rats fed for 3 or 12 months with 2 or 20 mg/kg (1 or 10x the human dose of DIM) or 50 mg/kg (5-7x the maximal recommended dose of I3C) for 3 or 12 months; no significant differences between groups were found in blood chemistry or histology. Males receiving I3C had higher serum concentrations of 25-hydroxyvitamin D3. Long-term exposure to DIM produced no observable toxicity. Compared to I3C, DIM is a significantly less effective CYP-inducing agent in rats [173]. In healthy humans, the safety, tolerability, and pharmacokinetics of a unique dose (50, 100, 150, 200, and 300 mg) of BioResponse 3,3’-diindolylmethane (BR-DIM), a compound nutritional grade and absorption enhancement were assessed. A unique dose of fewer than 200 mg of BR-DIM is considerably tolerated. However, at the 300-mg dose, were reports of nausea, headache, and vomiting [172]. In addition, no significant side effects are reported in administering DIM and I3C with hormonal therapy to modify the effects of estrogen on papillomas [174]. So, more studies are needed that evaluate the safety, side effects, and toxicity in humans at different doses and periods; data reported in the articles mentioned above suggest that oral administration seems safe and non-toxic.

## Conclusion

Recently, actions have been taken to design newer and safer drugs for cancer treatment. According to the results of studies revised and discussed in this paper, we identify the following highlights: (i) The low solubility and poor bioavailability of I3C and DIM limit their use as a medical treatment, so it is necessary to increase the research regarding pharmaceutic forms that improve these parameters, (ii) I3C and DIM have pro-apoptotic and antiproliferative effects and inhibit cell growth in pre-clinical and clinical cancer assays (Tables 2 and 3), (iii) Since I3C and DIM derivatives such as 5,5′-Br2-DIM, the regulated cellular process associated with hallmarks of cancer, it could be essential to evaluate also these derivatives as anti-cancer agents, maybe in conjunct with DIM or I3C, and finally, (iv) The current evaluation of the anti-tumoral activities of DIM and I3C is generally based on mechanisms involving signalizing ways such as NF-κ B, Akt, Wnt, PI3K/Akt/mTOR, and AhR. Because of the information discussed in this article, currently, a need for studies to elucidate the mechanism of action, the effective doses, toxicity studies, and pharmacologic interaction to use them as a chemotherapeutic drug.

## Data Availability

Not Applicable.

## Abbreviations

DIM-C-pPhCl: 1,1-bis(3′-indolyl)-1-(4-chlorophenyl)-methane
DIM-C-Pyr-4: 1,1-bis(3′-indolyl)-1-(4-pyridyl)-methane
DIM-D: 1,1-bis(3′-indolyl)-1-(p-chlorophenyl)-methane
DIM-C-pPhOH: 1,1-bis(3′-indolyl)-1-(p-hydroxyphenyl) methane
DIM-C-pPhOCH3: 1,1-bis(3′-indolyl)-1-(p-methoxyphenyl) methane
LTr1: 2-(indol-3-ylmethyl)-3,3’-diindolylmethane
2-OHE1: 2-hydroxyestrone
DIM: 3,3’-diindolylmethane
ICZ: 5,11-dihydroindolo-[3,2-b]carbazole
5,5′-Br2-DIM: 5,5′-dibromo-3,3’-diindolylmethane
CTr: 5,6,11,12,17,18-hexahydrocyclonone[1,2-b:4,5-b′:7,8-b″]tri-indole
DMBA: 7,12-dimethylbenz[α]anthracene
ATF3: Activating transcription factor 3
AMPK: AMP-activated kinase
AhR: Aryl hydrocarbon receptor
AEG-1: Astrocyte elevated gene 1
BR-DIM: BioResponse 3,3’-diindolylmethane
CDKIs: CDK inhibitors
CIN: Cervical intraepithelial neoplasia
COUP-TFI: Chicken ovalbumin upstream promoter-transcription factor I
CDK: Cyclin-dependent kinases
Egr-1: Early growth response 1
EMT: Epithelial-to-mesenchymal transition
ER: Estrogen receptor
EROD: Ethoxyresorufin-O-deethylase
EC50: Half of the maximum growth
I3N: Indole-3-acetonitrile
I3C: Indole-3-carbinol
IFN-γ: Interferon-gamma levels
MDM2: Mouse double minute 2 homolog
NR4A1: Nuclear receptor 4A1
PPARγ: Peroxisome proliferator-activated receptor γ
ROS: Reactive oxygen species
RRP: Recurrent respiratory papillomatosis
SD: Sprague-Dawley
SOCE: Store Operated Calcium Entry
C-DIM: Symmetrical ring-substituted DIM analogs
TRAIL: Tumor necrosis factor-related apoptosis-inducing ligand
uPA: Urokinase-type plasminogen activator
VEGF: Vascular endothelial growth factor
XREs: Xenobiotic Responsive Elements
